# An Investigation of the Shortcomings of the CONSORT 2010 Statement for the Reporting of Group Sequential Randomised Controlled Trials: A Methodological Systematic Review

**DOI:** 10.1371/journal.pone.0141104

**Published:** 2015-11-03

**Authors:** Abigail Stevely, Munyaradzi Dimairo, Susan Todd, Steven A. Julious, Jonathan Nicholl, Daniel Hind, Cindy L. Cooper

**Affiliations:** 1 The Medical School, University of Sheffield, Sheffield, United Kingdom; 2 School of Health and Related Research, University of Sheffield, Sheffield, United Kingdom; 3 Department of Mathematics and Statistics, University of Reading, Reading, United Kingdom; Toronto Western Hospital, CANADA

## Abstract

**Background:**

It can be argued that adaptive designs are underused in clinical research. We have explored concerns related to inadequate reporting of such trials, which may influence their uptake. Through a careful examination of the literature, we evaluated the standards of reporting of group sequential (GS) randomised controlled trials, one form of a confirmatory adaptive design.

**Methods:**

We undertook a systematic review, by searching Ovid MEDLINE from the 1^st^ January 2001 to 23^rd^ September 2014, supplemented with trials from an audit study. We included parallel group, confirmatory, GS trials that were prospectively designed using a Frequentist approach. Eligible trials were examined for compliance in their reporting against the CONSORT 2010 checklist. In addition, as part of our evaluation, we developed a supplementary checklist to explicitly capture group sequential specific reporting aspects, and investigated how these are currently being reported.

**Results:**

Of the 284 screened trials, 68(24%) were eligible. Most trials were published in “*high impact*” peer-reviewed journals. Examination of trials established that 46(68%) were stopped early, predominantly either for futility or efficacy. Suboptimal reporting compliance was found in general items relating to: access to full trials protocols; methods to generate randomisation list(s); details of randomisation concealment, and its implementation. Benchmarking against the supplementary checklist, GS aspects were largely inadequately reported. Only 3(7%) trials which stopped early reported use of statistical bias correction. Moreover, 52(76%) trials failed to disclose methods used to minimise the risk of operational bias, due to the knowledge or leakage of interim results. Occurrence of changes to trial methods and outcomes could not be determined in most trials, due to inaccessible protocols and amendments.

**Discussion and Conclusions:**

There are issues with the reporting of GS trials, particularly those specific to the conduct of interim analyses. Suboptimal reporting of bias correction methods could potentially imply most GS trials stopping early are giving biased results of treatment effects. As a result, research consumers may question credibility of findings to change practice when trials are stopped early. These issues could be alleviated through a CONSORT extension. Assurance of scientific rigour through transparent adequate reporting is paramount to the credibility of findings from adaptive trials. Our systematic literature search was restricted to one database due to resource constraints.

## Introduction

Appropriate use of adaptive designs has the potential to improve efficiency in the conduct of randomised clinical trials (RCTs). However, this can involve considerable extra work and effort during their planning, implementation, and reporting [1]. One form of adaptive design, namely a group sequential design, has been used in confirmatory RCTs for a number of years, which makes their review timely [2]. Regulators have described a group sequential design as well understood [3]. The design may offer ethical and economic benefits by allowing early stopping of RCTs, for instance, for futility or efficacy as soon as there is sufficient evidence to answer the research question(s). Nevertheless, adaptive designs are generally underused in routine practice, in relation to importance given to them in statistical literature [4,5]–although their use seems to be improving [1,6,7].

Various initiatives have been undertaken to facilitate discussions, and to address some of the barriers to the use of adaptive designs: predominately from a pharmaceutical drug development perspective [1,4,8–14]. Recent publicly funded research, in the UK confirmatory setting, found some degree of conservatism towards the use of adaptive designs [15]. This appears to be influenced by, among others: concerns regarding robustness of adaptive designs in decision making, credibility of their findings to change medical practice, and fear of making wrong decisions when trials are stopped early; and some worry about potential introduction of operational bias during trial conduct [15]. It could be argued that one potential solution to alleviate these cited concerns, is assurance of the scientific rigour in the conduct of RCTs, through transparency and adequate reporting. Thus enabling consumers of research findings to make informed judgements regarding the quality of the research in front of them.

The CONSORT (CONsolidated Standards Of Reporting Trials) statement was first published in 1996, with the aim to enhance adequate reporting of RCTs, [16] and has since been revised in 2001 and 2010 [17,18]. There has been marked general improvement in the conduct and reporting of RCTs since the advent of the first CONSORT statement [19–21], although there are still some suboptimal areas requiring improvements [21,22]. Extensions to the CONSORT statement have since been made to accommodate other trial designs and hypotheses, such as: cluster RCTs, non-inferiority and equivalence trials, and pragmatic RCTs [23–25]. As of the 23^rd^ September 2014, at least 30 reporting related guidance documents were being developed, to enhance transparency in the reporting and conduct of studies [26].

Although the CONSORT 2010 statement has some general items relating to “*interim analyses*”, there is currently no CONSORT statement tailored for adaptive designs. Moreover, recent research suggested that the current reporting guidance framework, for adaptive designs, is inadequate for research consumers and policy makers to make informed judgements [15]. Some authors have recently suggested modifications to the CONSORT statement to accommodate various forms of adaptive designs [27,28]. However, although these proposals seem robust in capturing adaptive features, they were not informed by evidence on what is considered to be important by key research stakeholders. Hence, some cited concerns raised by researchers, decision makers and policymakers, and key aspects requiring improvement may have been overlooked [15]. For instance, the use of appropriate inference, to obtain unbiased or bias corrected trial results (point estimates, confidence intervals [CIs] and P-values) was previously neglected. This review therefore aims to:

- Assess reporting compliance of group sequential designs, in confirmatory RCTs, against the CONSORT 2010 statement and some researcher-led proposed modifications (see items in S1 Table);
- Investigate the shortcomings of the CONSORT 2010 statement in enhancing the reporting of group sequential RCTs;
- Find exemplars of well reported group sequential RCTs, which could be used as references by researchers.

## Methods

### Eligibility criteria

This review was restricted to parallel group RCTs, conducted in humans, with confirmatory objectives. Eligible for inclusion were RCTs, which conducted prospectively planned interim analyses, within the class of group sequential designs using the Frequentist approach; regardless of the nature of: the primary endpoint(s), the number of intervention arms, and therapeutic area. Bayesian designed group sequential RCTs were excluded. In addition, only eligible RCTs with accessible, full-text, peer-reviewed reports, in the English language were included for the final examination of compliance in reporting.

### Literature search

Inconsistencies in the indexing related to the reporting of RCTs that employ interim analyses, using group sequential methodology, made the systematic search more challenging. MD conducted a scoping exercise by searching MEDLINE with the assistance of an experienced systematic reviewer, in order to develop an efficient search strategy. This scoping exercise found one MeSH term “*Early Termination in Clinical Trials*” which could be used to index some group sequential RCTs. However, the drawback of this MeSH term is that it biases the findings, in favour of trials that were stopped early, which is also an outcome of interest. The review is focusing on reporting of trials regardless of their early stopping status, and the MeSH search term was also insensitive when used via Ovid MEDLINE. Hence, a free text search was employed of keywords often associated with group sequential methodology, such as: “*group sequential*”, “*interim analys(i/e)s*”, “*stopping rule(s) or boundar(y/ies)*”, “*interim monitoring*”, “*early stopping or termination*” and “*accumulating data or information*”. Other more general terms such as: “*halted*”, “*closed*”, “*closure*”, “*independent data monitoring committee*” and “*data monitoring and safety board*” were excluded because they resulted in a very high number of irrelevant reports making the review impractical within time and resources constraints.

**Fig 1:**
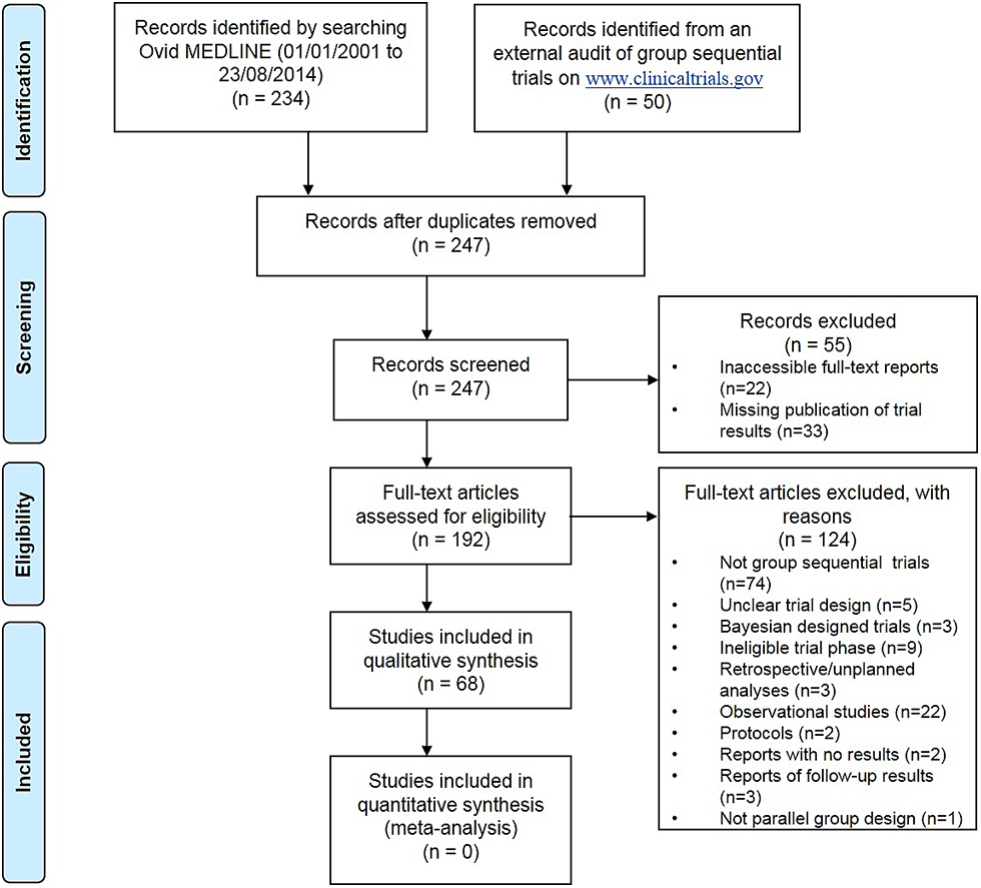
A modified PRISMA flowchart of the review process.

The search was used in combination with additional eligibility filters, namely publication type (*clinical trials*, *phase III*), check tags (*humans*, *full-text available*, *English language*) and publication year (*1* ^*st*^ *January 2001 to 23* ^*rd*^ *September 2014*). The final search combined independent searches with a Boolean operator “*OR*”. MD implemented the final search on the 23^rd^ September 2014 by searching Ovid MEDLINE. Systematic search was supplemented with some known group sequential RCTs, retrieved from an external audit study of adaptive designs on ClinicalTrials.gov [29] (see Fig 1). Duplicate records were checked and identified for exclusion based on the title, first author, and year of publication.

### Eligibility screening and quality control

Two reviewers (*AS*, *MD*) working independently, screened trial reports for eligibility, extracted characteristics of eligible trials and examined compliance in their reporting. Compliance was assessed against the CONSORT 2010 checklist items, and some proposed modifications. These captured issues such as: the use of appropriate statistical methods for early stopping bias correction, mechanisms put in place to minimise operational bias (due to the leakage or knowledge of interim results), access to prior interim results, rationale for choosing a group sequential RCT (with any other add-on planned adaptations), and discussion of the lessons learned and value of using a group sequential design, to help the planning of future trials (see S1 Table). Accessible additional related reports, such as protocols and other prior publications, were also used to assess compliance in reporting. All discrepancies were reviewed and rectified in agreement between the two independent reviewers (*AS*, *MD*). Study investigators were contacted for clarification where possible and necessary.

### Outcome measures

The primary outcome of the review is to establish compliance in reporting of the CONSORT 2010 checklist items, as well as the additional dimensions of interest specific to group sequential RCTs. This was subjectively examined and agreed upon by the two independent reviewers (*AS*, *MD*), according to a predefined classification system of completeness: “*absent*”, “*totally complete*”, “*partially complete*”, “*cannot access*” and “*not applicable*”. This was then used to compute the number and proportion of RCTs meeting total and at least partial, compliance in reporting criteria for each checklist item. Furthermore, a global measure of the number and proportion of checklist items meeting total and at least partial compliance criteria was calculated.

### Statistical analysis and reporting

The reporting of this review is in accordance with the PRISMA guidance [30,31]. Protocol registration correspondence is accessible (see S1 Appendix). Descriptive summary statistics including numbers (proportions) and median (IQR: Interquartile Range) for categorical and continuous data, respectively, were used to assess compliance in reporting. Clustered stacked bar charts and forest plots were used to aid visual interpretation. Two-sided 95% CIs around proportions were computed using the Wilson Score method [32]. Fisher’s exact test was used to explore differences in proportions between subgroups of interest, and estimates are presented as risk ratio (RR), with associated 95% CIs.

A global measure of compliance in reporting, based on the number and proportion of checklist items meeting a certain completeness criterion, was also employed. In addition, bootstrap methods [33] were used with 10 000 replicates, to compute the median difference, or median ratio (95% CI), of the total number of checklist items meeting certain reporting compliance criteria between subgroups. This approach was used to explore whether the publication journal’s CONSORT endorsement policy (yes or no), and publication period (pre- or post-publication of the CONSORT 2010 statement), was associated with improved compliance in reporting. The latter was used to explore the impact of the CONSORT 2010 statement in enhancing reporting. Comparability of compliance in reporting, between the standard CONSORT and researcher-led proposed items, was descriptive without any significance testing. Raw data are publicly accessible (see S1 Dataset).

## Results

### Eligibility screening

A total of 284 RCTs were screened for eligibility: of which, 234 were retrieved by searching the Ovid MEDLINE, and 50 were from an external audit study. Of these 284, 68(24%) peer-reviewed publications reporting main findings were eligible for examination of compliance in reporting. Reasons for exclusion and details of the screening process are shown on Fig 1.

### Characteristics of reviewed group sequential RCTs

The majority of RCTs were published in “*high impact*” medical journals such as; *The New England Medical Journal*, *The Lancet Oncology*, *The American Society of Clinical Oncology*, and *The Journal of the American Medical Association*. The median (IQR) journal impact factor for the year 2013 to 2014 was 17.5 (6.6 to 30.4), and the maximum was 54.4. Eligible group sequential RCTs were predominantly in the disease area of oncology (76%). However, diverse therapeutic conditions under investigation included cardiovascular, respiratory and infectious diseases. The majority, 62(91%), of the RCTs investigated at least some form of pharmacological intervention, and 55(81%) were designed with two intervention arms, inclusive of the comparator arm. Forty-six (68%) of the publishing journals endorsed the CONSORT statement as part of their publication policy. Table 1 describes detailed characteristics of examined eligible group sequential RCTs, stratified by publication period (pre- and post-publication of the CONSORT 2010 statement).

**Table 1:** Characteristics of eligible reviewed RCTs.

Variable	Scoring	Publication period		Total
		2001–2010	2011–2014	
		(n = 34)	(n = 34)	(n = 68)
Funder/sponsor	Private	16(47%)	19(56%)	35(51%)
	Public	8(24%)	11(32%)	19(28%)
	Private and Public	4(12%)	4(12%)	8(12%)
	None/independent	1(3%)	0(0%)	1(1%)
	Undisclosed	5(15%)	0(0%)	5(7%)
Nature of primary outcome(s)	Time-to-event	23(68%)	28(82%)	51(75%)
	Binary	6(18%)	3(9%)	9(13%)
	Continuous	3(9%)	3(9%)	6(9%)
	Binary and continuous	1(3%)	0(0%)	1(1%)
	Binary and time-to-event	1(3%)	0(0%)	1(1%)
Number of intervention arms	2	26(76%)	29(85%)	55(81%)
	3	6(18%)	3(9%)	9(13%)
	4	1(3%)	1(3%)	2(3%)
	5 or 6	1(3%)	1(3%)	2(3%)
Therapeutic area	Oncology	28(82%)	24(71%)	52(76%)
	HIV/AIDS	3(9%)	0(0%)	3(4%)
	Cardiac	0(0%)	2(6%)	2(3%)
	Musculoskeletal	1(3%)	1(3%)	2(3%)
	Optical	0(0%)	2(6%)	2(3%)
	Stroke	0(0%)	1(3%)	1(1%)
	Respiratory	1(3%)	0(0%)	1(1%)
	Diabetes	0(0%)	1(3%)	1(1%)
	Multiple Sclerosis	1(3%)	0(0%)	1(1%)
	Degenerative	0(0%)	1(3%)	1(1%)
	Epilepsy	0(0%)	1(3%)	1(1%)
	Kidney	0(0%)	1(3%)	1(1%)
Journal CONSORT endorsement status	No	13(38%)	9(26%)	22(32%)
	Yes	21(62%)	25(74%)	46(68%)
Publishing journal	The Lancet Oncology	3(9%)	9(26%)	12(18%)
	The New England Journal of Medicine	5(15%)	7(21%)	12(18%)
	American Society of Clinical Oncology	8(24%)	4(12%)	12(18%)
	Annals of Oncology	3(9%)	2(6%)	5(7%)
	The Journal of the American Medical Association	1(3%)	4(12%)	5(7%)
	Breast Cancer Research Treatment	2(6%)	1(3%)	3(4%)
	Journal of Clinical Oncology	2(6%)	1(3%)	3(4%)
	The Lancet	1(3%)	1(3%)	2(3%)
	The American Academy of Ophthalmology	0(0%)	2(6%)	2(3%)
	Arthritis and Rheumatology	0(0%)	1(3%)	1(1%)
	British Journal of Surgery	1(3%)	0(0%)	1(1%)
	Clinical Breast Cancer	0(0%)	1(3%)	1(1%)
	Clinical Cancer Research	1(3%)	0(0%)	1(1%)
	European Journal of Cancer	0(0%)	1(3%)	1(1%)
	HIV Clinical Trials	1(3%)	0(0%)	1(1%)
	Journal of the National Cancer Institute	1(3%)	0(0%)	1(1%)
	Journal of Urology	1(3%)	0(0%)	1(1%)
	Journal of the National Cancer Institute	1(3%)	0(0%)	1(1%)
	Nutrition	1(3%)	0(0%)	1(1%)
	Radiotherapy and Oncology	1(3%)	0(0%)	1(1%)
	The Journal of Infectious Diseases	1(3%)	0(0%)	1(1%)
Type of intervention	Drug	29(85%)	30(88%)	59(87%)
	Dietary	1(3%)	1(3%)	2(3%)
	Device	0(0%)	1(3%)	1(1%)
	Physiological	1(3%)	0(0%)	1(1%)
	Radiotherapy	1(3%)	0(0%)	1(1%)
	Drug and radiotherapy	0(0%)	1(3%)	1(1%)
	Drug and dietary	1(3%)	0(0%)	1(1%)
	Surgical	1(3%)	0(0%)	1(1%)
	Vaccine	0(0%)	1(3%)	1(1%)
Class of intervention	Pharmacological	30(88%)	32(94%)	62(91%)
	Non-pharmacological	4(12%)	2(6%)	6(9%)
Stage of reporting	Interim analysis	25(74%)	22(65%)	47(69%)
	Final analysis	7(21%)	6(18%)	13(19%)
	Unplanned interim analysis	2(6%)	6(18%)	8(12%)
Number of planned interims	1	16(47%)	12(35%)	28(41%)
	2	9(26%)	14(41%)	23(34%)
	3	3(9%)	2(6%)	5(7%)
	4	0(0%)	4(12%)	4(6%)
	5 or 7	3(9%)	0(0%)	3(4%)
	Undisclosed	3(9%)	2(6%)	5(7%)
Trials stopped early	No	11(32%)	9(26%)	20(29%)
	Yes	22(65%)	24(71%)	46(68%)
	No, but interim arm discontinued at interim	1(3%)	1(3%)	2(3%)
Reasons for early stopping (N = 46)	Futility	12(55%)	10(42%)	22(48%)
	Efficacy	5(23%)	5(21%)	10(22%)
	Safety	1(5%)	1(4%)	2(4%)
	Futility and safety	0(0%)	5(21%)	5(11%)
	Poor recruitment and/or financial	3(14%)	3(13%)	6(13%)
	Futility and external information	1(5%)	0(0%)	1(2%)
Planned stopping criteria	Undisclosed	16(47%)	6(18%)	22(32%)
	Futility or efficacy	8(24%)	12(35%)	20(29%)
	Futility	3(9%)	6(18%)	9(13%)
	Efficacy	0(0%)	6(18%)	6(9%)
	Efficacy or safety	3(9%)	1(3%)	4(6%)
	Futility or efficacy or safety	1(3%)	3(9%)	4(6%)
	Non-inferiority	2(6%)	0(0%)	2(3%)
	Safety	1(3%)	0(0%)	1(1%)
Planned total sample size	Min to Max	160–8028	100–15000	100–15000
	Median(IQR)	604(350–1071)	784(428–1200)	724(357–1155)

### Reporting of the universal CONSORT 2010 checklist items

**Fig 2:**
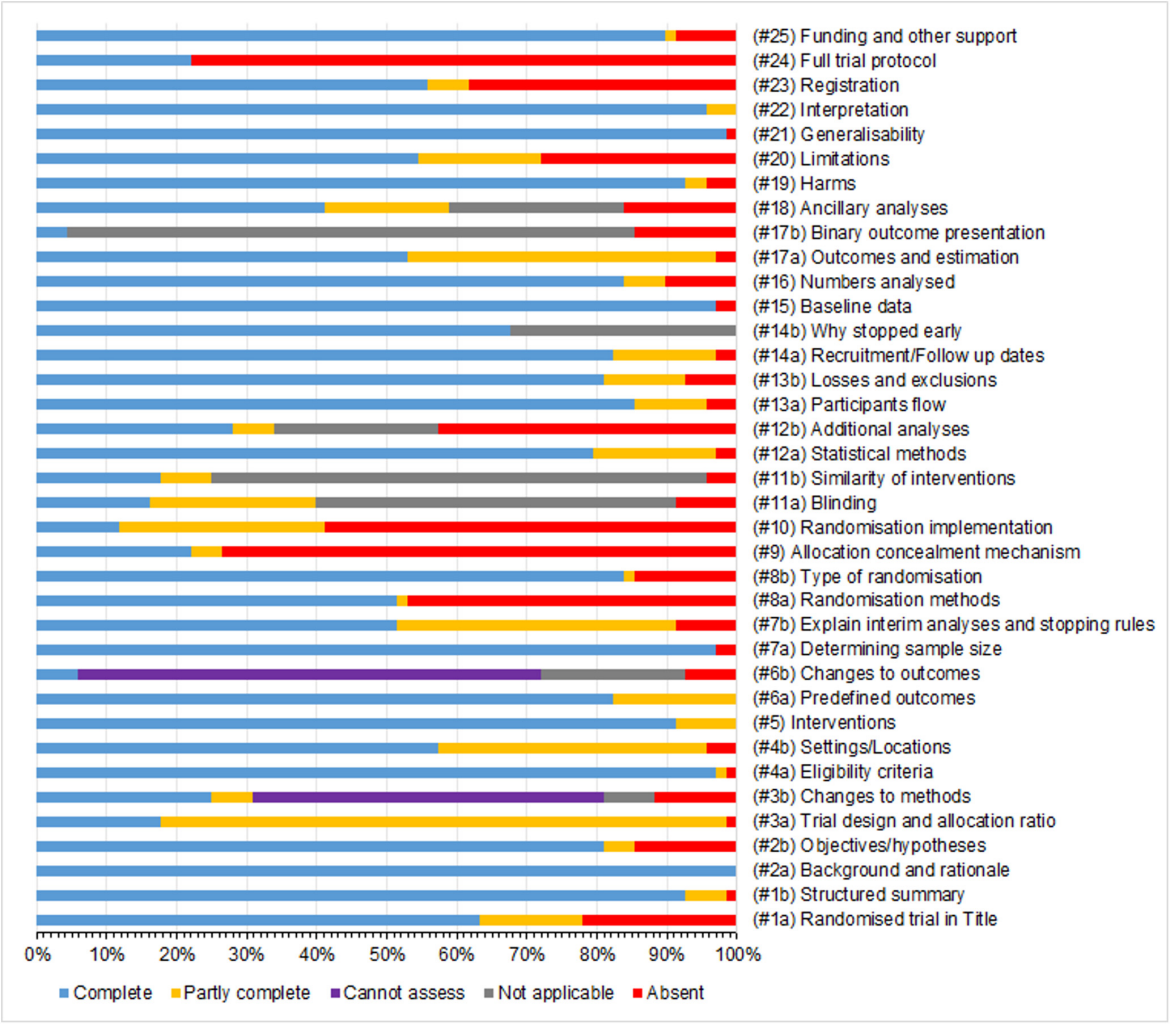
Clustered stacked bar charts of compliance in the reporting of general CONSORT 2010 checklist items.

**Fig 3:**
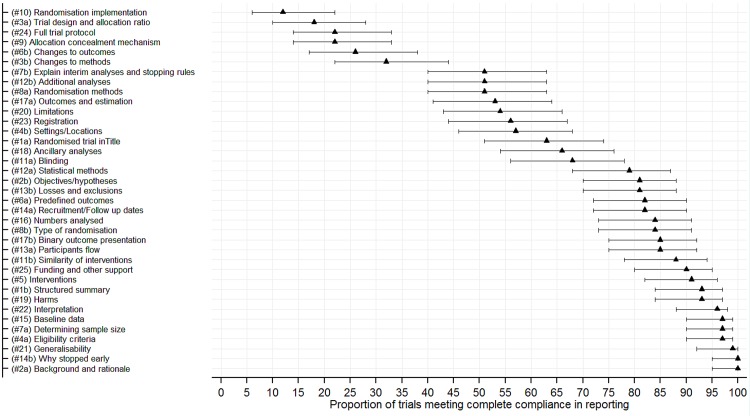
Forest plot of the proportion of trials meeting total completeness in the reporting of general CONSORT 2010 checklist items.

**Fig 4:**
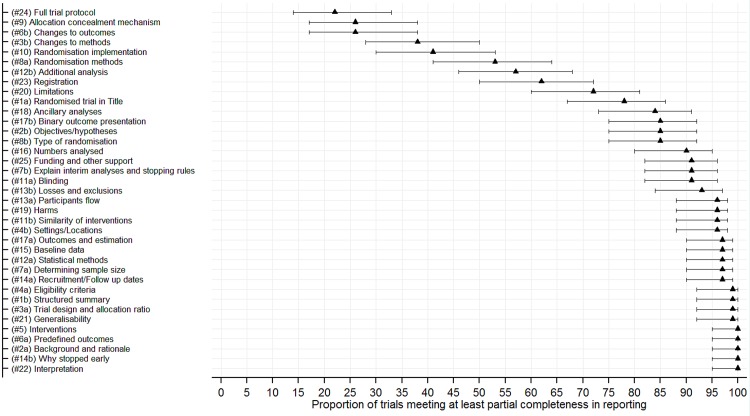
Forest plot of the proportion of trials meeting at least partial completeness in the reporting of general CONSORT 2010 checklist items.

Fig 2 shows a clustered bar chart of compliance in reporting against the CONSORT 2010 checklist items. Additional summary data are provided (see S2 Table). The median proportions (IQR) of RCTs meeting complete and at least partial compliance in reporting criteria of checklist items was 81% (53% to 91%) and 93% (78% to 97%), and a minimum of 12% and 22%, respectively. Figs 3 and 4 are forest plots showing the proportions of RCTs meeting total and at least partial completeness compliance criteria, with associated 95% CIs, respectively.

Suboptimal reporting was observed among checklist items relating to: the disclosure and access to full trial protocols 53(53%), methods used to generate the randomisation list(s) 32(47%), details of randomisation concealment 50(74%) and implementation of randomisation 40(59%), details of additional analysis 29(43%) and disclosure of trial registration information 26(38%). Furthermore, changes to methods and outcomes could not be assessed in 34(50%) and 45(66%) RCTs, respectively, due to inaccessible protocols and related amendments for most RCTs. Aspects relating to the trial design were partially reported in 55(81%) RCTs. However, most other checklist items were well reported.

Of the 37 CONSORT checklist items, the median number (proportion) [IQR] that were completely reported was 26(70%) [24(65%) to 28(76%)], and a minimum of 15(41%). However, this distribution was 30(81%) [29(78%) to 32(87%)], and a minimum of 24(65%) for checklist items that met partial compliance criterion. The median number (proportion) [95% CI] of items that met complete compliance increased by 2 (5%) [1(1%) to 4(10%); P = 0.009] post-publication of the CONSORT 2010 statement. The median difference (proportion) [95% CI] in items that met complete compliance in favour of journals that endorse the CONSORT statement as part of their publication policy was 1.5(4.1%) [-0.3(-0.9%) to 3.3(9.0%); P = 0.112].

### Reporting of group sequential specific checklist items and proposed modifications

**Fig 5:**
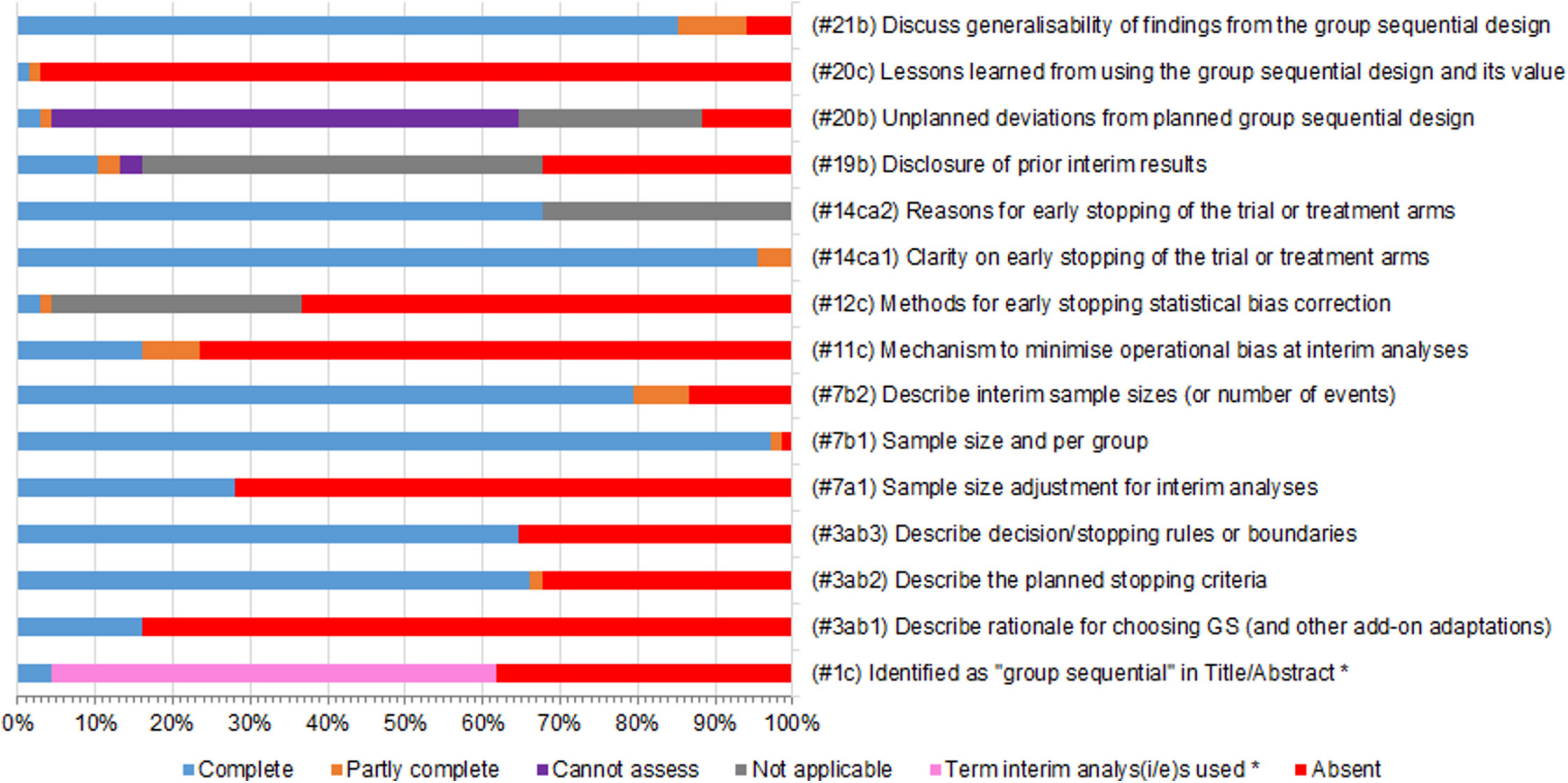
Clustered stacked bar charts of compliance in the reporting of group sequential-specific items. Only items marked (#3ab2), (#3ab3), (#7b1), (#7b2), (#14ca1), and (#14ca2) are partly or fully covered in the current CONSORT checklist. GS: Group Sequential.

Most items relating to group sequential aspects were poorly reported (see Fig 5). Additional summary data are provided (see S1 Table). Only 3(4%) RCTs were identifiable by the term “*group sequential*” in the Title or Abstract. An additional 39(57%) were identifiable by the terms “*interim analyses*” or “*interim analysis*”. The rationale for choosing a group sequential design (with any other add-on forms of trial adaptation) was only explained in 11(16%) RCTs.

Just 11(16%) RCTs adequately reported the mechanism used to minimise operational bias due to the knowledge or leakage of the interim results; 7 of these cited relevant prior publications. Of the 33 RCTs that were reporting interim results after the first interim, 9(27%) reported or disclosed prior interim results. Only 3 RCTs reported unplanned deviations from planned group sequential design and its potential implications on the findings. However, unplanned deviations could not be assessed in 41(60%) RCTs due to inaccessibility of protocols and associated amendments. Only 2 RCTs described the lessons learned from using the group sequential design, and its value in helping the planning of future group sequential RCTs.

The following aspects of a group sequential design were deemed to be adequately reported: description of the total (and per group) sample size, the planned number of interims and associated interim sample sizes (or number of events), and clarification on whether the RCT was stopped early with reasons where applicable. In addition, planned stopping criteria and rules or boundaries were deemed fairly reported. These particular interim analyses related items are well covered in the CONSORT 2010 checklist.

#### Early stopping of trials or treatment arms

Of the 68 RCTs, it was found that 46(68%) were stopped early; predominantly for futility, 61% (28/46), and efficacy, 22% (10/46) (see Table 1). The proportion of RCTs stopped early for any reasons before and after 2010 appeared to be similar; 22(65%) versus 24(71%), respectively: RR (95% CI, P-value); 0.92(0.66 to 1.27, P = 0.796). Of the 22 RCTs which were not stopped early, 6(27%) had multiple intervention arms. Of these 6 RCTs, 2 had discontinued one intervention arm at previous interim analyses. In 46 RCTs that were stopped early, the median (IQR) of the distribution of the proportion of interim sample size (or observed interim events) at the time of trial stopping relative to the planned was 65% (50% to 85%), and a minimum of 19%.

#### Type of planned stopping criteria

Stopping criteria and rules or boundaries planned at the design stage, were unreported in 22(32%) and 24(35%) of RCTs, respectively. Of the RCTs that reported planned stopping criteria and/or stopping boundaries, 11(16%) cited additional relevant information accessible in the form of prior publications or protocols. Thirty-three (49%) RCTs were planned with at least some form of futility early stopping criteria; 9(13%) for futility only, 20(29%) for either futility or efficacy, and 4(6%) for futility, efficacy or safety.

#### Type of planned stopping rules or boundaries

Twenty-four (35%) RCTs did not disclose the stopping rules or boundaries used. Of the 44(65%) RCTs that reported stopping rules or boundaries, the most frequently used stopping boundaries were: 15(34%) Lan-DeMets (LD) [34] error spending function mimicking O’Brien-Fleming (OBF) type properties [35], and 12(27%) OBF. Other stopping boundaries or rules that were rarely used were: Pocock [36]; Haybittle-Peto [37,38]; Pampallona and Tsiatis [39], in combination with LD error spending function of the OBF type; Pampallona and Tsiatis [39]; Gamma family (γ = -8) [40]; Rho family (ρ = 3) [41], in combination with OBF; Wang and Tsiatis (shape parameter of 0) [42], in combination with OBF; Fleming [43], in combination with LD error spending function of the OBF type; Whitehead’s double triangular test [44]; conditional power based; and reported in terms of number of events or hazard ratios.

#### Number of planned interim analyses and stage of reporting

The majority (75%) of RCTs were planned with either one or two interim analyses. There were very few RCTs planned with large numbers of interim analyses (see Table 1). Only 5(7%) RCTs did not report the number of planned interim analyses. 55(81%) RCTs were reporting interim results; of which, 47(69%) were as intended. Poor recruitment and/or financially related issues were the main reasons for reporting unplanned interim results in the remaining 8(12%) RCTs.

#### Early stopping statistical bias correction

Of the 46 RCTs that were stopped early, only 3(7%) reported the use of appropriate statistical methods for bias correction of point estimates of the intervention effect, and associated CIs and P-values; 2 of these were stopped early for futility and/or safety. Only 1 of the 10 RCTs that were stopped early for efficacy reported the use of bias corrected statistical methods to conduct inference.

### Exemplars to enhance reporting of group sequential RCTs

Although there were no publications that met complete compliance on all checklist items, we found exemplars of group sequential RCTs that reported most items adequately [45–47]. The PRIMO trial is an exemplar that provided a comprehensive rationale of choosing, and detailed description of a group sequential design incorporating sample size re-estimation used [48,49]. Some publications described aspects of the randomisation process, allocation concealment and its implementation, which were found to be problematically reported in most group sequential RCTs, better than others [47,50]. A further exemplar reported detailed description of protocol changes [51]. Another useful exemplar gave a description, and graphical representation, of prior interim trends of the intervention effect, and explored the trend using regression methods [52].

Roger et al [45] gave a clear description of an exact statistical method, used to obtain unbiased inference following early stopping of group sequential RCTs:

“*The study design is based on a group-sequential test procedure with pre-planned analyses after 220*, *320 and 428 patients meeting one of the off-study criteria*. *An alpha-spending approach as suggested by Lan and DeMets* [34] *with an O’Brien/Fleming-like alpha spending function was used to define the test boundaries of the group-sequential procedure*. *The primary analysis regarding OS uses a Cox Proportional Hazard Model with treatment and prognosis groups as predictor variables to calculate the Z score needed for the group- sequential procedure*. *Stagewise ordering was used to compute the unbiased median estimate and confidence limits for the prognosis-group-adjusted hazard rates* [53].”

One exemplar explained deviations from the planned interim analyses and the implications on their findings [54]. Finally, two RCTs discussed lessons learned from, value of, and implications of using a group sequential approach [55,56].

## Discussion

### Main findings

We found a significant number of confirmatory RCTs that employ interim analyses using group sequential methods, particularly in oncology, although the therapeutic areas of application appear to be diverse. Moreover, the majority of the group sequential RCTs were published in “*high impact*” peer-reviewed medical journals, and were often stopped early, predominantly for futility or efficacy.

We found inadequate reporting of CONSORT checklist items relating to the disclosure and access to trial protocols, methods used to generate the randomisation list(s), details of randomisation concealment and its implementation, and trial design aspects. Most concerning, is the lack of access to full trial protocols, with related amendments, in the public domain, for most of the examined group sequential RCTs. Hence, we could not ascertain the completeness in reporting of other key checklist items, such as changes to methods and outcomes. Despite this, compliance in reporting of most CONSORT 2010 checklist items appeared to be very high.

In general, additional important features of group sequential RCTs are poorly reported. These encompass; rationale for choosing a group sequential design with any other add-on planned adaptations, mechanisms put in place to minimise operational bias due to the knowledge of interim results, lessons learned from using a group sequential design and its value, and clarification on whether sample size was adjusted for interim analyses. Most importantly, we found suboptimal reporting of the use of appropriate statistical methods for early stopping bias correction of point estimates, with associated CIs and P-values.

Reporting of planned stopping criteria, and stopping rules or boundaries employed, is still unsatisfactory, despite the fact that aspects are covered in the CONSORT 2010 statement. Nonetheless, some aspects of interim analyses, such as clarification of early stopping with reasons where applicable, description of; interim sample sizes (or number of events), number of planned interim analyses and timing, were adequately reported.

### Interpretation of the findings

Our review findings are predominantly based upon group sequential RCTs, published in “*high impact*” medical journals, particularly in oncology. Hence, the general quality of compliance in reporting, may exaggerate what might be observed based on reports in other therapeutic areas, or lower impact journals for some checklist items [57–59]. For instance, suboptimal compliance to most checklist items has been reported in previous reviews in other therapeutic areas [58,59]. Regardless of the publishing journal’s impact factor, trial design and therapeutic area, suboptimal reporting of randomisation methods, and details of randomisation concealment and its implementation has been widely reported and is consistent with our findings [57–61]. Similar findings on these checklist items were also found in oncology, and moreover, inadequate reporting was associated with exaggerated, biased intervention effects [61].

Most importantly, our findings of very poor reporting and use, of statistical methods for bias correction for early stopping, are consistent with previous findings of a systematic review, focusing on trials which stopped early for benefit [62]. This dimension has been overlooked in some proposed modifications to the CONSORT 2010 statement [27].

We believe that our findings on areas requiring improvement provide a conservative picture, of the scale of the problem regarding compliance in reporting of group sequential specific aspects, which are vital for research consumers to make informed judgements about the quality of findings.

### Implications to practice

Our review uncovered group sequential RCTs published predominantly in “*high impact*” medical journals. To some extent, this may provide assurance to sceptical researchers, who may have concerns pertaining to poor receptiveness by journal editors and reviewers towards adaptive designs, when RCTs are stopped early [15]. In contrast, suboptimal reporting of appropriate statistical methods for early stopping bias correction, may influence some research consumers, who are aware of the phenomenon of exaggerated intervention effects when a naïve statistical approach is used, to consider findings from group sequential RCTs with a degree of scepticism. Research consumers, such as clinicians and regulators, may be reluctant to accept these findings in order to change medical practice, when trials are stopped early coupled with failure to implement bias correction, and poor communication and reporting of the corrective actions taken [15,62].

The phenomenon of exaggeration of the intervention effects in group sequential RCTs, following early stopping when a naïve statistical approach is used, has been widely debated and highlighted [62–69]. Although much attention has been paid to group sequential RCTs that are stopping early for benefit [62,63,66,68], the consequences could be similar when trials are stopped early for futility, since the evidence can be used to withdraw intervention(s) already in the care pathway. More so, it could be argued that the consequences on future evidence synthesis, through meta-analysis, should be treated similarly regardless of the reasons for early stopping.

Although there does not exist a unique solution to early stopping correction of statistical bias, various statistical procedures have been proposed [67,70–76], and it appears that these methods are rarely implemented by statisticians in routine practice. What is unclear, is the extent of the impact of statistical bias correction, on the results and decision making of these group sequential RCTs, particularly those that are stopped early. Some examples of RCTs, where interpretation of findings changed after bias correction, have been reported [62]. In contrast, one case study reported consistent interpretation of findings from using a naïve statistical approach, and bias correction under various methods [67]. The lack of knowledge of the impact that inaccurate analyses has on decision making among statisticians, lack of awareness of bias adjustment methods and unfamiliarity with mainstream statistical software(s) offering options to implement these procedures, could be contributing to their poor uptake. The scale of this problem in routine practice has been honestly articulated [67]:

“*One purpose of this article is to right a wrong I committed about a decade ago as part of the group reporting the results of The Randomized Aldactone Evaluation Study (RALES)*. *… The trial stopped early after crossing an O’Brien–Fleming-like boundary (calculated through a Lan–DeMets spending function)*, *and we reported the results as we saw them; we corrected neither the p-value nor the effect size for having stopped early*. *I have consoled myself for this lapse because I knew that other trials that had stopped early had also failed to correct the observed results for having stopped early*.”

The increasing use of add-on trial adaptations (such as sample size re-estimation and treatment selection in multiple arm studies), within the group sequential framework, adds more complexities requiring more transparency in the design, conduct and reporting of these trials, beyond the checklist items covered by the CONSORT 2010 statements [27].

Our findings support initiatives for mandatory publication of, not only full trial protocols, but also all related protocol amendments. Assessing the quality of reporting of some key aspects of RCTs, proved challenging without access to these important trial documents, most imperative for complex adaptive designs. We absolutely concur with previous findings, that assessment of methodological quality should be based on evaluation of both protocols and publications [61].

Interim analyses heighten anxiety among some research consumers, due to the potential for introducing operational bias to the conduct of RCTs, thereby undermining the scientific integrity, credibility and validity of the findings. The potential introduction of operational bias, due to the leaking or knowledge of interim results in adaptive RCTs, has been well described [13,77]. However, the extent and impact of operational bias on the findings and decision making in routine practice is less well-understood [27]. Therefore, it is imperative to report mechanisms put in place, to minimise and/or control for operational bias such as; who conducted the interim analyses, how data was transferred and results were communicated, who were the stakeholders in interim decision-making process, and how decisions were made. Although it is difficult to prevent indirect inference of interim results, due to decisions made following an interim analysis; careful planning, implementation, and optimal reporting of the mechanisms put in place, may go a long way in alleviating research consumers’ worries about operational bias.

Our findings of poor reporting or inaccessibility of, prior interim results at the interim reporting, or point of early stopping, could hinder the ability of consumers of research findings, to assess trends of the direction of the treatment effects and the potential effect of population drift. Although it is challenging to distinguish between natural and population drift induced by operation bias, access to prior interim results may help research consumers to make their own informed judgements, and alleviate some of the cited concerns.

### Recommendations to practice

Based upon our findings and desire to improve: the reproducibility and planning of future trials, the acceptability of findings from group sequential RCTs and to reduce waste in trials research [78]; we recommend urgent cross-disciplinary extension to the CONSORT 2010 statement, specifically aspects tailored for group sequential designs, most of which are consistent with recent suggestions [27]. We believe this would improve the reporting of group sequential RCTs and any other add-on adaptations considered. These aspects may encompass, but are not limited to:

▪ The use of the term “*group sequential*” or at least “*interim analys(i/e)s*” in the Title or Abstract for easy identification of group sequential RCTs for future systematic reviews and meta-analyses;
▪ Provision for the rationale of choosing a group sequential design and any add-on adaptations used;
▪ Detailed description of the mechanisms put in place to minimise (and control for) operational bias due to the leaking or knowledge of the interim results;
▪ Clarification on the use of, and description of, appropriate statistical methods to obtain unbiased results (point estimates, CIs, and P-values) when trials are stopped early. This should also encompass description of appropriate statistical inferential methods used to account for add-on trial adaptations where appropriate;
▪ Disclosure of prior interim results on the primary endpoint(s) and related decisions made. Summary tables or figures showing trends in the results or citation of previous related publications may suffice;
▪ Discussion of the lessons learned by using a group sequential design, and any other add-on adaptations where appropriate, and its value, with implications to aid the planning of future trials;
▪ Discussion of the deviations from the planned group sequential design and any other add-on adaptations, and their implications on generalisability of the findings.

We also encourage better reporting of group sequential aspects, which are already covered briefly in the CONSORT 2010 statement, such as planned stopping criteria and rules or boundaries used. Finally, more attention by journal editors and reviewers, and researchers, should be given to complete reporting of trial design, including any add-on adaptations; methods to generate randomisation list(s); randomisation concealment, and its implementation; and disclosure of full trial protocols (with related amendments).

### Strengths and limitations

The need for this review has been based upon concerns raised by researchers, decision makers and policymakers regarding robustness and credibility of adaptive designs in decision making, to change practice when trials are stopped early [15]. We provided a comprehensive examination of compliance in the reporting of group sequential RCTs, utilising all accessible publication related reports using an improved classification system. Furthermore, the systematic search was supplemented with known group sequential RCTs from another source, and provided exemplars which could be used to enhance adequate reporting. However, one of the major limitations is that the literature search was restricted to Ovid MEDLINE due to resources and time limitations. Moreover, group sequential RCTs are not systematically indexed in the titles and abstracts and their key characteristics are poorly reported. Hence, our searching of Ovid MEDLINE could have missed a significant number of eligible trials. Inaccessibility of trials protocols and associated amendments hampered the assessment of some key CONSORT checklist items, such as changes to methods and outcomes. Finally, exploration of factors associated with suboptimal reporting was out of scope of this research.

## Conclusions

There are issues with suboptimal reporting of key group sequential specific characteristics, such as disclosure of the use of appropriate adjusted inferential methods following early stopping. Suboptimal reporting of bias correction methods could potentially imply most group sequential trials stopping early are giving biased results of treatment effects. These issues may partly explain the cited concerns about robustness and acceptability of the methodology to change practice, when trials are stopped early. These concerns could be alleviated by modifications to the CONSORT 2010 statement. Assurance of scientific rigour through transparent and adequate reporting of RCTs is paramount to the acceptability of findings from adaptive designs in general. There is an urgent need for CONSORT statement(s) tailored for adaptive design(s). Improvements in the reporting of general CONSORT checklist items relating to access to trial protocols, trial design aspects, methods to generate randomisation list(s), concealment of the randomisation and its implementation is required.

We hope our findings will enlighten researchers on potential ways to address some of the concerns we highlighted. We hope our results will also inform the CONSORT working group regarding the need for modifications to enhance the conduct and reporting of group sequential RCTs and adaptive trials in general.

## Supporting Information

S1 Appendix PROSPERO protocol registration correspondence. (PDF)

S1 Dataset Raw data on completeness in reporting of reviewed RCTs. (XLSX)

S1 Table Summary data of compliance in the reporting of group sequential specific and researcher-proposed modified checklist items. (DOCX)

S2 Table Summary data of compliance in the reporting of general CONSORT 2010 checklist items. (DOCX)

## Abbreviations

CI: Confidence Interval
CONSORT: CONsolidated Standards Of Reporting Trials
GS: Group Sequential
IQR: Interquartile Range
LD: Lan-DeMets
RCT: Randomised Controlled Trial
RR: Risk Ratio
OBF: O’Brien-Fleming
PRISMA: Preferred Reporting Items for Systematic reviews and Meta-Analyses
