# Supplementary material for: An Investigation of the Shortcomings of the CONSORT 2010 Statement for the Reporting of Group Sequential Randomised Controlled Trials: A Methodological Systematic Review
Source: PLoS One. 2015 Nov 3;10(11):e0141104. doi: 10.1371/journal.pone.0141104 (PMC4631356; doi:10.1371/journal.pone.0141104)
Supplement: S1 Table — (DOCX) [file pone.0141104.s003.docx]

| **Checklist item** | **Completeness in reporting of group sequential specific aspects** | | | | | |
| --- | --- | --- | --- | --- | --- | --- |
|  | **Absent** | **Complete** | **Partly complete** | **Cannot assess** | **Not applicable** | **Interim analys(i/e)s** |
| (#1c) Identified as “*group sequential*” in Title/Abstract * § | 26(38%) | 3(4%) | - | - | - | 39(57%) |
| (#3ab1) Describe the rationale for choosing the group sequential design (and other add-on adaptations) § | 57(84%) | 11(16%) | - | - | - | - |
| (#3ab2) Describe the stopping criteria employed | 20(29%) | 47(69%) † | 1(1%) | - | - | - |
| (#3ab3) Describe the stopping rules employed | 25(37%) | 43(63%) † | - | - | - | - |
| (#7a1) Sample size adjusted for interim analyses § | 49(72%) | 19(28%) | - | - | - | - |
| (#7b1) Sample size and per group | 1(1%) | 66(97%) | 1(1%) | - | - | - |
| (#7b2) Describe interim sample sizes (or number of events) | 9(13%) | 54(79%) | 5(7%) | - | - | - |
| (#11c) Measures to minimise operational bias due to leakage or knowledge of interim results § | 52(76%) | 11(16%) ϙ | 5(7%) | - | - | - |
| (#12c) Describe use of statistical methods for early stopping bias correction § | 44(65%) | 2(3%) | 1(1%) | - | 21(31%) | - |
| (#14ca1) Clarification on whether the trial (or treatment arms §) were stopped early stopping | - | 65(96%) | 3(4%) | - | - | - |
| (#14ca2) Reasons for early stopping of the trial (or treatment arms §) | - | 46(68%) | - | - | 22(32%) | - |
| (#19b) Describe prior interim results when applicable § | 22(32%) | 7(10%) | 2(3%) | 2(3%) | 35(51%) | - |
| (#20b) Described unplanned deviations to the planned group sequential design § | 8(12%) | 2(3%) | 1(1%) | 41(60%) | 16(24%) | - |
| (#20c) Discuss lessons learned and the value of the group sequential design § | 66(97%) | 1(1%) | 1(1%) | - | - | - |
| (#21b) Discuss generalisability of the results from the group sequential design § | 4(6%) | 58(85%) | 6(9%) | - | - | - |

† 11 trials described stopping boundaries/rules and/or stopping rules elsewhere in cited material

ϙ 7 trials described measures to minimise operational bias due to leakage or knowledge of interim results

* 39(57%) trials identified by the terms “*interim analysis*” or “*interim analyses*”

§ Marked items (or parts) are researcher-led proposed modifications
