# Supplementary material for: An Investigation of the Shortcomings of the CONSORT 2010 Statement for the Reporting of Group Sequential Randomised Controlled Trials: A Methodological Systematic Review
Source: PLoS One. 2015 Nov 3;10(11):e0141104. doi: 10.1371/journal.pone.0141104 (PMC4631356; doi:10.1371/journal.pone.0141104)
Supplement: S2 Table — (DOCX) [file pone.0141104.s004.docx]

| **CONSORT checklist item** | **Completeness in reporting of general CONSORT items** | | | | | |
| --- | --- | --- | --- | --- | --- | --- |
|  | **Partial/Complete**  **/Not applicable** | **Absent** | **Complete** | **Partly complete** | **Cannot assess** | **Not Applicable** |
| (#1a) Randomised trial in title | 53(78%) | 15(22%) | 43(63%) | 10(15%) | - | - |
| (#1b) Structured summary | 67(99%) | 1(1%) | 63(93%) | 4(6%) | - | - |
| (#2a) Background and rationale | 68(100%) | - | 68(100%) | - | - | - |
| (#2b) Objectives/hypotheses | 58(85%) | 10(15%) | 55(81%) | 3(4%) | - | - |
| (#3a) Trial design and allocation ratio | 67(99%) | 1(1%) | 12(18%) | 55(81%) | - | - |
| (#3b) Changes to methods | 26(38%) | 8(12%) | 17(25%) | 4(6%) | 34(50%) | 5(7%) |
| (#4a) Eligibility criteria | 67(99%) | 1(1%) | 66(97%) | 1(1%) | - | - |
| (#4b Settings/Locations | 65(96%) | 3(4%) | 39(57%) | 26(38%) | - | - |
| (#5) Interventions | 68(100%) | - | 62(91%) | 6(9%) | - | - |
| (#6a) Predefined outcomes | 68(100%) | - | 56(82%) | 12(18%) | - | - |
| (#6b) Changes to outcomes | 23(34%) | 5(7%) | 4(6%) | - | 45(66%) | 14(21%) |
| (#7a) Determining sample size | 66(97%) | 2(3%) | 66(97%) | - | - | - |
| (#7b) Explain interim analysis and stopping | 62(91%) | 6(9%) | 35(51%) | 27(40%) | - | - |
| (#8a) Randomisation methods | 36(52%) | 32(47%) | 35(51%) | 1(1%) | - |  |
| (#8b) Type of randomisation | 58(85%) | 10(15%) | 57(84%) | 1(1%) | - | - |
| (#9) Allocation concealment mechanism | 18(26%) | 50(74%) | 15(22%) | 3(4%) | - | - |
| (#10) Randomisation implementation | 28(41%) | 40(59%) | 8(12%) | 20(29%) | - | - |
| (#11a) Blinding | 51(91%) | 6(9%) | 11(16%) | 16(24%) | - | 35(51%) |
| (#11b) Similarity of interventions | 65(96%) | 3(4%) | 12(18%) | 5(7%) | - | 48(71%) |
| (#12a) Statistical methods | 66(97%) | 2(3%) | 54(79%) | 12(18%) | - | - |
| (#12b) Additional analyses | 39(57%) | 29(43%) | 19(28%) | 4(6%) | - | 16(24%) |
| (#13a) Participants flow | 65(96%) | 3(4%) | 58(85%) | 7(10%) | - | - |
| (#13b) Losses and exclusions | 63(93%) | 5(7%) | 55(81%) | 8(12%) | - | - |
| (#14a) Recruitment/Follow up dates | 66(97%) | 2(3%) | 56(82%) | 10(15%) | - | - |
| (#14b) Why stopped early | 68(100%) | - | 46(68%) | - | - | 22(32%) |
| (#15) Baseline data | 66(97%) | 2(3%) | 66(97%) | - | - | - |
| (#16) Numbers analysed | 61(90%) | 7(10%) | 57(84%) | 4(6%) | - | - |
| (#17a) Outcomes and estimation | 66(97%) | 2(3%) | 36(53%) | 30(44%) | - | - |
| (#17b) Binary outcome presentation | 58(85%) | 10(15%) | 3(4%) | - | - | 55(81%) |
| (#18) Ancillary analyses | 57(89%) | 11(16%) | 28(41%) | 12(18%) | - | 17(25%) |
| (#19) Harms | 65(96%) | 3(4%) | 63(93%) | 2(3%) | - | - |
| (#20) Limitations | 48(72%) | 19(28%) | 37(54%) | 12(18%) | - | - |
| (#21) Generalisability | 67(99%) | 1(1%) | 67(99%) | - | - | - |
| (#22) Interpretation | 68(100%) | - | 65(96%) | 3(4%) | - | - |
| (#23) Registration | 42(62%) | 26(38%) | 38(56%) | 4(6%) | - | - |
| (#24) Full trial protocol | 15(22%) | 53(78%) | 15(22%) | - | - | - |
| (#25) Funding and other support | 62(91%) | 6(9%) | 61(90%) | 1(1%) | - | - |
