# Supplementary material for: An Investigation of the Shortcomings of the CONSORT 2010 Statement for the Reporting of Group Sequential Randomised Controlled Trials: A Methodological Systematic Review
Source: PLoS One. 2015 Nov 3;10(11):e0141104. doi: 10.1371/journal.pone.0141104 (PMC4631356; doi:10.1371/journal.pone.0141104)
Supplement: S1 Appendix — (PDF) [file pone.0141104.s001.pdf]

The University Of Sheffield.

Munya Dimairo <m.dimairo@sheffield.ac.uk>

PROSPERO Registration message [15809]

CRD-REGISTER <crd-register@york.ac.uk> 6 January 2015 at 11:33
Reply-To: crd-register <crd-register@york.ac.uk>
To: "m.dimairo@sheffield.ac.uk" <m.dimairo@sheffield.ac.uk>

Dear Dr Dimairo

Thank you for submitting details of your systematic review *Reporting issues in group sequential randomised controlled trials: a systematic review protocol of published journal articles* to the PROSPERO register.

We regret that the information you have provided fails to meet the requirements for inclusion. Specifically, this is a methodology review which has no directly health related outcomes.

We hope that this will not discourage you from registering your next systematic review at the protocol stage, with PROSPERO.

Yours sincerely

James Christie

PROSPERO Administrator
Centre for Reviews and Dissemination
University of York
York YO10 5DD
t: +44 (0) 1904 321040
f: +44 (0) 1904 321041
e: CRD-register@york.ac.uk
www.york.ac.uk/inst/crd

CRD is part of the National Institute for Health Research and is a department of the University of York.

Email disclaimer: http://www.york.ac.uk/docs/disclaimer/email.htm
